# Patient-reported urinary incontinence following stereotactic body radiation therapy (SBRT) for clinically localized prostate cancer

**DOI:** 10.1186/1748-717X-9-148

**Published:** 2014-06-26

**Authors:** Leonard N Chen, Simeng Suy, Hongkun Wang, Aditi Bhagat, Jennifer A Woo, Rudy A Moures, Joy S Kim, Thomas M Yung, Siyuan Lei, Brian T Collins, Keith Kowalczyk, Anatoly Dritschilo, John H Lynch, Sean P Collins

**Affiliations:** 1 Department of Radiation Medicine, Georgetown University Hospital, 3800 Reservoir Road, N W, Washington, DC 20007, USA; 2 Biostatistics and Bioinformatics, Georgetown University Medical Center, Washington, DC 20057, USA; 3 Department of Urology, Georgetown University Hospital, Washington, DC 20007, USA

**Keywords:** Prostate cancer, SBRT, Urinary incontinence, Expanded prostate index composite, EPIC, CyberKnife, Quality of life

## Abstract

**Purpose:**

Urinary incontinence (UI) following prostate radiotherapy is a rare toxicity that adversely affects a patient’s quality of life. This study sought to evaluate the incidence of UI following stereotactic body radiation therapy (SBRT) for prostate cancer.

**Methods:**

Between February, 2008 and October, 2010, 204 men with clinically localized prostate cancer were treated definitively with SBRT at Georgetown University Hospital. Patients were treated to 35–36.25 Gray (Gy) in 5 fractions delivered with the CyberKnife (Accuray). UI was assessed via the Expanded Prostate Index Composite (EPIC)-26.

**Results:**

Baseline UI was common with 4.4%, 1.0% and 3.4% of patients reporting leaking > 1 time per day, frequent dribbling and pad usage, respectively. Three year post treatment, 5.7%, 6.4% and 10.8% of patients reported UI based on leaking > 1 time per day, frequent dribbling and pad usage, respectively. Average EPIC UI summary scores showed an acute transient decline at one month post-SBRT then a second a gradual decline over the next three years. The proportion of men feeling that their UI was a moderate to big problem increased from 1% at baseline to 6.4% at three years post-SBRT.

**Conclusions:**

Prostate SBRT was well tolerated with UI rates comparable to conventionally fractionated radiotherapy and brachytherapy. More than 90% of men who were pad-free prior to treatment remained pad-free three years following treatment. Less than 10% of men felt post-treatment UI was a moderate to big problem at any time point following treatment. Longer term follow-up is needed to confirm late effects.

## Background

Involuntary leakage of urine is a common problem of aging. Urinary incontinence (UI) varies in frequency and severity, making it clinically challenging to define. In men greater than sixty five years old, the prevalence of UI may be as high is 31% [1]. UI is caused by an overactive bladder (urge incontinence) and/or poor urethral sphincter function (stress incontinence) [2]. Aging, comorbidities, obesity, benign prostatic hypertrophy, prostate cancer and its treatments may increase the risk of UI [3]. Urinary continence recovery following radical prostatectomy is variable with 5-30% of patients still requiring pads one year after surgery [4,5]. The incidence of UI after external beam radiation therapy or brachytherapy varies considerably with a reported range of 10% to 30% [6-9]. The incidence of UI is dependent on the definition utilized [10] and on the manner of data collection (i.e. patient or physician reported) [11]. Post-treatment UI develops months to years after radiation therapy without recovery [8,9] and may adversely affect a patient’s sexual function and general quality of life [12]. Bother from UI varies based on its severity [13,14]. UI is commonly refractory to treatment [15] and the daily usage of pads is a burden for patients and caregivers [16].

Clinical data suggest that hypofractionated radiation therapy may be radiobiologically favorable to smaller fraction sizes in prostate cancer radiotherapy [17]. The α/β for prostate cancer may be as low as 1.5 Gy (Gray) [17]. If the α/β for prostate cancer is less than 3 Gy, which is generally the value accepted for late urinary complications, the linear-quadratic model predicts that delivering large radiation fraction sizes will result in improved local control with a similar rate of urinary complications. Early data from trials of limited hypofractionation (fraction sizes from 2.5 to 3.5 Gy) revealed that such regimens are effective without undue urinary toxicity [18].

Stereotactic body radiation therapy (SBRT) uses even larger daily fractions of radiation (7–9 Gy) to take further advantage of this postulated radiobiological advantage. Emerging clinical data suggest that this approach may provide similar clinical outcomes as other radiation modalities with high rates of biochemical control and low rates of grade 3 and higher toxicities [19-25]. Based on patient preference for a shorter treatment course, SBRT utilization is likely to increase as long as toxicity is acceptable. Here, we present our institutional patient-reported urinary incontinence rates following SBRT for clinically localized prostate cancer.

## Methods

### Patient selection

Patients eligible for study inclusion had histologically-confirmed prostate cancer treated per our institutional protocol. Prospectively collected quality of life (QoL) data for all patients included in our institutional database were retrospectively analyzed with Georgetown University Internal Review Board (IRB) approval.

### SBRT treatment planning and delivery

SBRT treatment planning and delivery were conducted as previously described [22,26]. Four gold markers were placed into the prostate. Several days after marker placement, patients underwent magnetic resonance (MR) imaging followed shortly thereafter by a computed tomography (CT) scan. Fused CT and MR images were used for treatment planning. The clinical target volume (CTV) included the prostate and the proximal seminal vesicles. The planning target volume (PTV) equaled the CTV expanded 3 mm posteriorly and 5 mm in all other dimensions. The prescription dose was 35–36.25 Gy to the PTV delivered in five fractions of 7–7.25 Gy corresponding to a tumor equivalent dose in 2-Gy fractions (EQD2) of approximately 85–90 Gy assuming an alpha/ beta ratio of 1.5. The bladder and membranous urethra were contoured and evaluated with dose-volume histogram analysis during treatment planning using Multiplan (Accuray Inc., Sunnyvale, CA) inverse treatment planning as previously defined. The dose-volume histogram (DVH) goals were for < 50% membranous urethra and < 5 cc of the bladder receiving 37 Gy. To minimize the risk of local recurrence, the dose to the prostatic urethra was not constrained [27]. Target position was verified multiple times during each treatment using paired, orthogonal x-ray images [28].

### Follow-up and statistical analysis

Patients completed the Short Form-12 Health Survey (SF-12) [29], the American Urological Association Symptom Index (AUA) [30] and Expanded Prostate Cancer Index Composite (EPIC)-26 [31] before treatment and during routine follow-up visits one month after the completion of SBRT, every 3 months for the first year and then every 6 months for the second and third years. UI was assessed via the urinary incontinence domain of the Expanded Prostate Index Composite (EPIC)-26 [31]. The EPIC-26 UI domain includes three questions related to function (Questions 1–3 of the EPIC-26) and one question related to bother (Question 4 of the EPIC-26). The functions assessed included UI frequency, urinary control and pad usage [32]. For each EPIC question, the responses were grouped into three to four clinically relevant categories. UI rates were defined using three separate commonly employed definitions: leaking > one time per day, frequent dribbling and daily pad usage [8]. To statistically compare changes between time points, the levels of responses were assigned a score and the significance of the mean changes in the scores was assessed by paired *t* test.

EPIC summary scores for the UI domain range from 0–100 with lower values representing worsening UI. The minimally important difference (MID) in EPIC score was defined as a change of one-half standard deviation (SD) from the baseline [33]. The EPIC UI domain summary scores were stratified to three levels of severity as previously described [34]: severe (0–49), moderate (50–69) and mild (70–100). Multiple logistic regression with backward elimination was used in the multivariate analysis to search for possible predicting factors for UI. The endpoint for this analysis was the EPIC UI subdomain score at 3 years post-SBRT. Baseline characteristics including age, prostate volume, α_1A_ inhibitor usage, and AUA scores were included as variables in the logistic regression model.

## Results

From February 2008 to October 2010, 204 prostate cancer patients were treated per our institutional SBRT monotherapy protocol (Table 1). The median follow-up was 3.9 years. They were ethnically diverse with 45.6% being of non-Caucasian ancestry and a median age of 69 years (range, 48–90 years). Obesity and comorbidities were common. 50% of patients had moderate to severe lower urinary tract symptoms prior to treatment (baseline AUA ≥ 8) with a median baseline AUA of 7.5 (Table 2). The median prostate volume was 39 (11.6-138.7) cc and 7.8% had prior procedures for benign prostatic hyperplasia (BPH). 27.9% of patients utilized alpha-antagonists prior to SBRT. By D’Amico classification, 81 patients were low-, 106 intermediate-, and 17 high-risk. Twenty nine patients (14.2%) also received androgen deprivation therapy (ADT) with median duration of 3 months (range, 3–24 mon). 88.2% of the patients were treated with 36.25 Gy in five 7.25 Gy fractions.

**Table 1:** Baseline patient characteristics and treatment

		**Patients *(N=204)***
**Age (y/o)**	Median 69 (48~90)	
	Age ≤ 60	12.7%
	60 < Age ≤ 70	46.6%
	Age > 70	40.7%
**Race**	White	54.4%
	Black	38.7%
	Other	7.8%
**Charlson Comorbidity Index**	CCI=0	65.2%
	CCI=1	21.1%
	CCI≥2	13.7%
**Body Mass Index (BMI)**	Median 27.60 (15.02-44.96)	
	BMI ≥ 30	30.5%
**Partner Status**	Married or Partnered	76.0%
	Not Partnered	24.0%
**Employment Status**	Working	48.0%
	Not Working	52.0%
**Median Prostate Volume (cc)**	Median 39 (11.6-138.7) cc	
**Procedure for BPH**		7.8%
**α**_**1A **_**inhibitor usage**		27.9%
**Risk Groups (D’Amico’s)**	Low	39.7%
	Intermediate	52.0%
	High	8.3%
**ADT**		14.2%
**SBRT Dose**	36.25 Gy	88.2%
	35 Gy	11.8%

**Table 2:** Pre-treatment Quality of Life (QOL) scores

	**(n=204)**		
**Baseline AUA Score**	% Patients		
**0-7 (mild)**	50.0%		
**8-19 (moderate)**	43.6%		
**≥ 20 (Severe)**	6.4%		
**Baseline SF-12 Score**	Mean (Range)	SD	
**PCS (Physical Health Score)**	49.9 (15.6-64.4)	8.76	
**MCS (Mental Health Score)**	56.6 (27.2-69.5)	6.71	
**Baseline EPIC-26 Incontinence Score**	Mean (Range)	SD	MID
**Bother**	92.5 (25-100)	14.79	7.4
**Summary**	92.3 (18.8-100)	13.99	7.0

**Figure 1:**
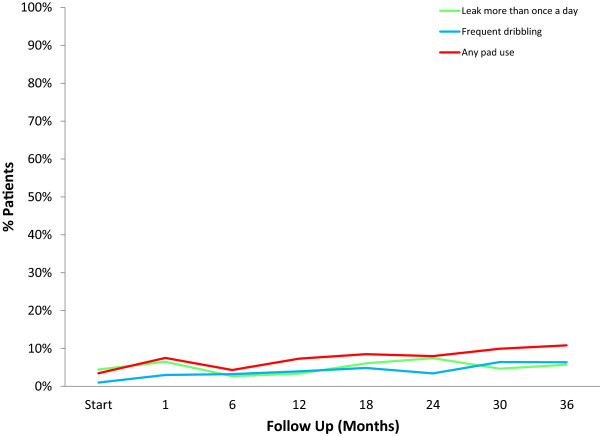
**Differences in rates of urinary incontinence based on definition.** Percentage of patients with leakage > 1 time a day, frequent dribbling or daily pad usage.

Baseline UI was common in our patients. Prior to treatment, 4.4% of the patients reporting leaking once per day or more and 1% reported frequent dribbling or no control at all (Table 3, Figure 1). Unexpectedly, 3.5% of patients reported using one or more pads per a day prior to SBRT (Table 3, Figure 1). At three year post treatment, 5.7%, 6.4% and 10.8% of patients reported incontinence based on the definitions of leaking > one time per day, frequent dribbling and pad usage, respectively (Table 3, Figure 1). However, only 1.9% reported no control of urination and only 4.5% reported using more than one pad per a day (Table 3). The increase in pad usage was unlikely solely due to aging, as the mean age of pad-using patients at 36 months (73.8 y/o) was not statistically different from non-pad using patients (71.3 y/o) (*p* = 0.106).

**Table 3:** Urinary Incontinence following SBRT for prostate cancer: patient-reported responses to EPIC-26 questions 1 (frequency of leakage), 2 (urinary control), 3 (pad usage), 4a (dripping or leaking urine) and UI domain scores

	**Start**	**1 M**	**6 M**	**12 M**	**18 M**	**24 M**	**30 M**	**36 M**
***N=***	***204***	***200***	***186***	***178***	***165***	***175***	***171***	***157***
**Frequency of leakage**								
**Never leak**	79.3%	69.5%	69.9%	69.1%	69.7%	68.0%	65.5%	64.3%
**Leak ≤ 1 time/day**	16.3%	24.0%	27.4%	27.5%	24.2%	24.6%	29.8%	29.9%
**Leak >1 time/day**	4.4%	6.5%	2.7%	3.4%	6.1%	7.4%	4.7%	5.7%
**Urinary Control**								
**Total Control**	72.9%	61.8%	61.8%	54.8%	57.6%	65.1%	59.1%	57.3%
**Occasional dribbling**	26.1%	35.2%	34.9%	41.2%	37.0%	31.4%	34.5%	36.3%
**Frequent dribbling**	0.0%	2.0%	1.6%	1.7%	3.0%	3.4%	5.8%	4.5%
**No control**	1.0%	1.0%	1.6%	2.3%	1.8%	0.0%	0.6%	1.9%
**Pad Usage**								
**No pads**	96.6%	92.5%	95.7%	92.7%	91.5%	92.0%	90.1%	89.2%
**1 pad/day**	3.0%	5.5%	3.2%	5.1%	6.1%	5.1%	6.4%	6.3%
**≥ 2 pads/day**	0.5%	2.0%	1.1%	2.2%	2.4%	2.9%	3.5%	4.5%
**Bother-dripping/leaking**								
**No problem**	75.9%	62.9%	68.3%	61.8%	64.2%	60.6%	60.8%	58.0%
**Small problem**	23.2%	34.5%	30.1%	34.8%	29.7%	33.7%	34.5%	35.7%
**Mod-Big problem**	1.0%	2.5%	1.6%	3.4%	6.1%	5.7%	4.7%	6.4%
**UI Domain**								
**Mild (70-100)**	90.1%	85.5%	86.6%	84.8%	81.8%	85.1%	83.6%	84.7%
**Moderate (50-69)**	7.4%	11.0%	11.8%	12.4%	14.5%	9.1%	11.1%	8.3%
**Severe (0-49)**	2.5%	3.5%	1.6%	2.8%	3.6%	5.7%	5.3%	7.0%

**Figure 2:**
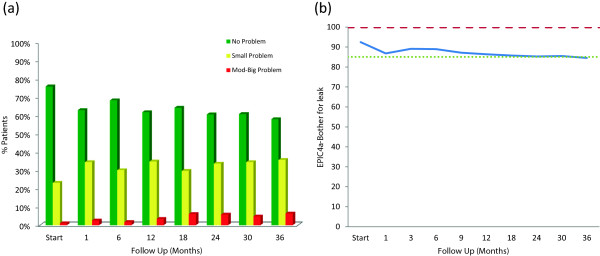
**Bother with dripping or leaking at baseline and following SBRT for prostate cancer- Question 4 of the EPIC-26. (a)** Patients were stratified to three groups: no problem, very small-small problem and moderate-big problem. The percentage of patients in each group at each time point is depicted in the bar chart. **(b)** Average EPIC bother with dripping or leaking scores at baseline and following SBRT for prostate cancer. Thresholds for clinically significant changes in scores (½ standard deviation above and below the baseline) are marked with dashed lines. EPIC scores range from 0–100 with higher values representing a more favorable health-related QOL.

Treatment-related bother may be more important to an individual patient than treatment-related dysfunction. At baseline, 24.2% of our cohort reported some level of bother due to urinary dripping or leaking with 1.0% of the patients feeling it was a moderate to big problem (Table 3, Figure 2a). The baseline UI bother score is shown in Table 2 and mean changes in EPIC UI bother scores from baseline to 3 years of follow-up are shown in Table 4. The mean EPIC UI bother score was 92.5 at baseline (Table 2). UI bother increased following treatment with the mean score decreasing to 86.8 at 1 month post-treatment (mean change, −5.69) (*p* < 0.0001) (Table 4, Figure 2b). However, only 2.5% of patients felt that that was a moderate to big problem at 1 month following treatment (Table 3, Figure 2a). Although UI bother improved quickly, a second late worsening in UI bother was observed with the mean UI bother score decreasing to 84.55 at 36 months (mean change from baseline, −7.93) (p < 0.0001) (Table 4, Figure 2b). Only the decline at 36 months met the threshold for clinically significant change (MID =7.4). The proportion of men feeling that their UI was a moderate to big problem increased to 6.4% at three years post-SBRT (Table 3, Figure 2a).

**Table 4:** Changes in urinary incontinence bother and urinary incontinence summary scores following SBRT for prostate cancer

	**1 Month Post-Treatment**			**3 Month Post-Treatment**			**12 Month Post-Treatment**			**24 Month Post-Treatment**			**36 Month Post-Treatment**		
	**Change from Baseline**	**SD**	***P***	**Change from Baseline**	**SD**	***P***	**Change from Baseline**	**SD**	***P***	**Change from Baseline**	**SD**	***P***	**Change from Baseline**	**SD**	***P***
**UI Bother**	-5.69	19.65	< 0.0001	-3.35	18.74	0.007	-6.11	20.08	< 0.0001	-7.2	22.93	< 0.0001	-7.93	22.56	< 0.0001
**UI Summary**	-4.09	16.74	< 0.0001	-1.84	14.99	0.065	-4.77	16.35	< 0.0001	-4.69	18.47	< 0.001	-6.46	19.45	< 0.0001

**Figure 3:**
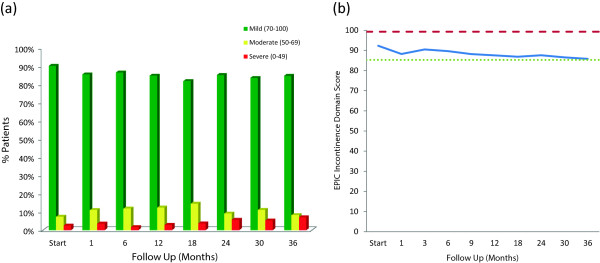
**EPIC Urinary Incontinence Domain. (a)** Patients were stratified to three groups: severe (0–49), moderate (50–69) and mild (70–100). **(b)** Average EPIC urinary incontinence domain scores at baseline and following SBRT for prostate cancer. Thresholds for clinically significant changes in scores (½ standard deviation above and below the baseline) are marked with dashed lines. EPIC scores range from 0–100 with higher values representing a more favorable health-related QOL.

There is no universally-accepted definition for UI and commonly employed definitions based on responses to individual questions do not fully assess the clinical impact of the problem (i.e., symptom, dysfunction and bother). Domain summary scores more comprehensively assess the clinical impact of UI on the patient [31]. The baseline EPIC UI summary score is shown in Table 2 and mean changes in EPIC UI summary scores from baseline to 3 years of follow-up are shown in Table 4. At baseline, 10% of our cohort had moderate to severe UI (Table 3, Figure 3a). The mean EPIC UI summary score was 92.3 at baseline (Table 2). The EPIC UI summary score declined acutely at 1 month post-SBRT (mean change, −4.09) (Table 5, Figure 3b). However, only 14.5% of patients had moderate to severe UI (Table 3, Figure 3a). The EPIC UI summary score returned to near baseline by three months post-SBRT (mean change from baseline, −1.84) (Table 4, Figure 3b). This acute decline was statistically (*p* < 0.0001) but not clinically significant (MID = 7). Average EPIC UI summary scores showed a second late protracted decline over the next three years (Table 4, Figure 3b). At three years post-treatment, the mean summary score decreased from a baseline of 92.31 to 85.85 (mean change from baseline at 36 months, −6.46) (Table 4). This change was statistically (p < 0.0001) but of borderline clinical significance (MID = 7). The proportion of men with moderate to severe UI increased to 15.3% at three years post-SBRT (Table 3, Figure 3a).

**Table 5:** Impact of baseline characteristics on EPIC-UI score three years post-SBRT

	**Model with age and prostate volume**			
	**Estimate**	**Standard error**	**t Value**	**Pr > |t|**
**Intercept**	111.57196	15.64089521	7.13	< 0.0001
**Age**	-0.254749	0.22871957	-1.11	0.2671
**Prostate Volume**	-0.198787	0.07516895	-2.64	0.0091

When modeled with EPIC UI scores at 3 years post-SBRT, age was not highly correlated with the UI outcome (p = 0.2671, Table 5). From a bivariate relationship, prostate volume and α_1A_ antagonist usage are highly associated with the outcome, although after adjusting for age, only the prostate volume was highly associated with UI score (p = 0.0091, Table 5). No other baseline patient characteristics were significantly associated with UI score at three years following SBRT.

## Discussion

Urinary incontinence following prostate cancer treatment is common [35] and an important quality of life issue [8]. A better understanding of the risk of UI following SBRT enables clinicians to provide more realistic expectations to patients as they weigh complex treatment options [36]. Currently, there is limited data on incidence of UI following SBRT for prostate cancer [22]. Previously, we reported a 10-15% risk of UI following SBRT. However, these findings relied on physician reported UI rates, which may under report the actual incidence of UI and provided no information on the associated bother [11]. In this study, we utilized the urinary incontinence domain of the EPIC-26 to comprehensively evaluate patient reported UI following SBRT [31].

The incidence of post treatment UI varies greatly depending on the definition utilized [10]. In the absence of a consensus definition of UI, direct comparison between treatment techniques remains difficult. The most commonly used definition of UI is daily pad usage [37]; however, even “pad free” men may experience periodic leakage. Likewise, men using only one pad per day may be using it due to frequent leakage or as a precaution [32,38-40]. Thus for this analysis, we evaluated the three commonly used definitions to assess the incidence and associated dysfunction. In addition, we examined the bother associated with UI (Table 6).

**Table 6:** Correlation between urinary incontinence bother and urinary incontinence definition at three years post-SBRT

	**No problem**	**Small problem**	**Moderate-big problem**
**Leak > Once/day**	0.0%	33.3%	66.7%
**Frequent dribbling**	20.0%	20.0%	60.0%
**Any pad usage**	5.9%	58.8%	35.3%
**EPIC UI summary “Severe” (0-49)**	0.0%	18.2%	81.8%

As in other radiation therapy series, our patients were elderly with poor baseline urinary function and a high prevalence of UI prior to treatment [8]. At one month post-treatment there was an acute increase in UI that resolved by three months post SBRT. This acute increase was likely secondary to transient cystitis/urethritis, which when moderate to severe may cause urge incontinence [41]. As observed previously following treatment with alternative radiation modalities [8,9,42,43], a second gradual increase in UI occurred from 3 months to the end of follow-up without recovery. Even so, by three years post-SBRT, only 6% of these patients reported leaking more than once per day and only 5% reported needing more than one pad per day. Despite the high biologically effective dose (BED) delivered by SBRT in this series, these results seem similar to conventionally fractionated EBRT, proton therapy and brachytherapy [8,44]. It seems to be that despite the fact that patients in this study are older and have more comorbidities, the incontinence rates compare favorably to radical prostatectomy [8].

The etiology of late UI following prostate cancer radiation therapy appears to be multi-factorial [35], and is likely due in part to aging and related comorbidities [42]. Prostate cancer itself may impair the integrity of the anatomic structures that maintain urinary continence. When compared to their peers without prostate cancer, even men who choose active surveillance are at increased risk of UI [37,45,46]. Previous studies of alternative radiation modalities have also reported that late toxicity following radiation therapy is associated with pretreatment factors related to BPH, such as a high AUA score [47,48] and a large prostate volume [49]. Post-RT TURP may increase the incidence of urinary incontinence [50]. In this series, late UI secondary to post-RT TURP was rare. The one patient who experienced this had a history of benign prostatic hypertrophy with a large prostate and two prior TURP procedures prior to receiving SBRT [22].

Compared with urinary function, bother may be a more accurate indicator of the impact of treatment on an individual patient’s quality of life. Defined as the degree of interference or annoyance caused by urinary incontinence, bother is dependent on an individual’s pretreatment function [51,52]. In general, previously continent men report higher rates of post-treatment bother. In this series of men, UI bother gradually increased during the first three years following SBRT treatment (Figure 2, Table 4). Two years following SBRT, 6% of men reported UI bother as a moderate to big problem. This change is comparable to that reported at 24 months with conventionally fractionated IMRT (5%), proton therapy (4%) and brachytherapy (6%) [8,44].

Commonly employed UI definitions based on answers to single questions do not fully assess the clinical impact of urinary incontinence (i.e., symptom, dysfunction and bother). Domain summary scores more comprehensively assess the clinical impact of UI on the patient [31]. Our EPIC UI summary domain outcomes appear similar to those previously reported for high dose conventionally fractionated intensity modulated radiation therapy (IMRT)/proton therapy and brachytherapy [8,9]. The mean urinary incontinence score change from baseline at 24 months was – 4.7. This change from baseline was statistically significant but not clinically significant. Importantly, this change is comparable to that seen at 24 months with IMRT, proton therapy and brachytherapy, − 5.1, −4.1 and −6.0 respectively [8,9].

## Conclusions

SBRT for clinically localized prostate cancer was well tolerated with UI rates comparable to conventionally fractionated external radiotherapy and brachytherapy. More than 90% of men who were pad-free prior to treatment remained pad-free three years following treatment. Less than 10% of men felt post-treatment UI was a moderate to big problem at any time point following treatment. Longer term follow-up is needed to confirm late effects.

### Consent

This retrospective review of prospectively collected data was approved by the Georgetown University Institutional Review Board.

## Abbreviations

ADT: Androgen deprivation therapy; AUA: American Urological Association; BED: Biologically effective dose; BPH: Benign prostatic hyperplasia; CT: Computed tomography; CTV: Clinical target volume; DVH: Dose-volume histogram; EQD2: Equivalent dose in 2-Gy fractions; EPIC: Expanded Prostate Index Composite; GTV: Gross target volume; Gy: Gray; IMRT: Intensity modulated radiation therapy; IRB: Internal Review Board; PTV: Planning target volume; QoL: Quality of life; MID: Minimally important difference; MR: Magnetic resonance; SD: Standard deviation; SF-12: Short Form-12 Health Survey; SBRT: Stereotactic body radiation therapy; UI: Urinary incontinence.

## Competing interests

SP Collins and BT Collins serve as clinical consultants to Accuray Inc. The Department of Radiation Medicine at Georgetown University Hospital receives a grant from Accuray to support a research coordinator. The other authors declare that they have no competing interests.

## Authors’ contributions

LC and SS are lead authors, who participated in data collection, data analysis, manuscript drafting, table/figure creation and manuscript revision. HW participated in data analysis and manuscript drafting, table/figure creation and manuscript revision. AB and JW aided in the quality of life data collection and maintained the patient database. RM and JK aided in the quality of life data collection and maintained the patient database. TY aided in clinical data collection. SL is the dosimetrist who developed the majority of patients’ treatment plans, and contributed to the dosimetric data analysis and interpretation. BC and KK participated in the design and coordination of the study. AD is a senior author who aided in drafting the manuscript. JL is a senior author who aided in drafting the manuscript. SC was the principal investigator who initially developed the concept of the study and the design, aided in data collection, drafted and revised the manuscript. All authors read and approved the final manuscript.
